# Letter from Dr. David Prince

**Published:** 1866-02

**Authors:** David Prince

**Affiliations:** 21 W. 12th St., New York


To Dr. N. S. Davis, *Ed. Chicago Med. Examiner:*

Dear Sir:—Upon the subject of ventilation, there is evidence that the world moves. The problem has been, how to secure the comfort of a home with the purity of outdoor air. It has been supposed that this was the utmost possible attainment. More than this has been achieved. It has been found practicable to make an artificial mountain air without its rarity.

Mr. A. S. LYMAN, a noted *inventor*, has, through 20 years of study and experiment, finally perfected an arrangement of chemical agents, so as to deprive the air of all its impurities and make it equal to that found above the influence of emanations from the surface of the earth.

The expedient consists in passing air over unslaked lime, which absorbs carbonic acid and some water and organic impurities, and elevates its temperature, giving it a tendency to rise. It then passes through fresh-burned charcoal, losing most of the impurities which the lime had not taken up. The purified air then ascends to the top of the apparatus, and, turning to descend, it passes over ice, which cools it and accelerates the current, which next passes out through orifices in which the current is divided up by wire screens, so as not to blow in one compact current. This apparatus is the head of a bed, or the back part of a desk or bookcase, or, on a larger scale, it is a part of a system of ventilation of a whole house.

When the apparatus is part of a bed, the sides of the bed are made high enough, either by curtains or the arrangement of the wooden framework, so that the cooled and purified air flowing in, at the head of the bed, does not flow immediately off upon the floor, but accumulates upon the patient or sleeper, covering and immersing him thus in a bath of cool pure air.

For the greatest benefit of this expedient, when applied to a desk, there should be a tight enclosure for the reader or writer to sit in, which becomes filled higher than the top of his head, so that, in the impure air of a city in a hot summer's day, he may sit in and breathe a chemically pure atmosphere, reduced to a temperature compatible with vigorous mental activity.

Other substances may be used for the purpose of taking impurities out of the air, but Mr. LYMAN, from numerous trials, has come to prefer fresh-burned lime, fresh-heated charcoal, and ice. In winter time, the ice is a less essential part of the ingredients, but in hot weather, the cooling of the air is a source of health and comfort which all can appreciate.

The principle of purification applies to the air of marshy regions occasioning ague, to general impurities of air, arising from all the innumerable kinds of decomposition in cities, favoring whatever epidemic may be impending, whether erysipelas, cholera, or dysentery, and to the air of crowded rooms, and the wards of hospitals generating typhus and typhoid fevers and imparting to wounds erysipelas, diffuse suppuration, and gangrene, and where the bone is involved in injury, ostitis and osteomyelitis; generally contaminating the blood with the poison absorbed with decomposing fluids, and involving the whole system in the low fever of pyæmia.

The infection of air contaminated with the decomposing organic exhalations from the lungs, or from pus and blood in a state of decomposition, is no less destructive to puerperal women who are very much in the condition of persons recently wounded, and who are subject to the same accidents of local and general poisoning through the contact of decomposing fluids and of air loaded with fœtid gases and floating cells and scales, ready, like spores, to produce their like wherever they may alight.

The device of Mr. LYMAN secures for patients a pure air to breathe, and in which to lie, but another *desideratum*, not yet fully realized, is an arrangement by which the expired air and other emanations of patients with infectious diseases shall be carried directly away without contaminating the air of the room in which they lie, thus affording protection to physicians and nurses who must sit or move about in the room.

It will be gratifying to the medical profession and to all philanthropists to know that Mr. LYMAN proposes to make his invention free of any charge for patent right to hospitals maintained for the benefit of the poor.

Some beds with the purifying attachment have been in operation, during the winter, at Bellevue Hospital, to the very great satisfaction of those who have watched the progress of the cases submitted to the influence of the purified air.

It is an old story in hot weather, in the progress of an epidemic, "If the weather would only change—if the wind would only blow from the North-west, we should have an improvement in the patients." It will certainly be a boon to mankind, and especially to all sick persons, to have the means of changing the weather, and, for the space of a few feet, of producing a North-west wind, as pure and as bracing as any that was ever manufactured in the laboratory of the heavens.

A new era in hospital construction will have dawned, when plans shall have been adopted to give them a purer atmosphere than exists in the free breezes outside. That this has been the desire of all who have investigated the subject, any one can see who reads a few pages, at random, upon what has been written upon hygiene during the last twenty years. I cannot do better than to quote a paragraph from Dr. KRACKOWIZER, from the *Bulletin of the New York Academy of Medicine*, vol. ii., 1865, p. 438.

"If, then, physicians look with a certain awe and resignation upon the ravages of pyæmia, it is not because they know less of its causes and of its nature than of other diseases, but because they see plainly how the cause might be removed, yet, under certain circumstances, are denied the means to prevent the disease.

"It is bad enough that the surgeon and the obstetrician find inherent difficulties in the treatment of wounds, in the management of abnormal labor, that they cannot help sometimes making mistakes in choosing the times for operations and in performing them; but it is discouraging to have to take an element into the calculation, in performing one's professional duty, that neither lies in the nature of the case, nor can be warded off by any degree of scientific acumen or professional skill.

"The preventive hygienic treatment goes before everything. If the sums spent all over the world for every sort of medicine in healing pyæmia—quinia included—could be accumulated in a fund wherewith to build a hospital in some part of the world, so perfect in its hygienic arrangements that pyæmia in it would be impossible, more would be done for diminishing its ravages, than by all the attempts to heal patients when they once are its victims."

Dr. Krackowizer truly says, "the *preventive* hygienic treatment goes before everything." According to Dr. Hammond (in his *Military Hygiene*, p. 426,) the amount of carbonic acid in the military hospitals which were found in the best condition was considerably above that in the outdoor air. While outside there was $\frac{37}{100}$ of a part in 1000 parts, there was in those in which there was the least attention paid to ventilation, $2\frac{11}{100}$ parts by volume in 1000 volumes of air.

Leblanc found in one of the wards of the Salpetriere, 8 parts of carbonic acid in 1000 by weight, or $5\frac{33}{100}$ by volume, in 1000 volumes.

Dr. Hammond expresses the opinion, that 10 parts of carbonic acid in 1000 volumes of air, renders it altogether unfit for respiration, and that a smaller amount must be unhealthy in proportion. But it is probable that the organic material proceeding from the lungs and cutaneous surface is far more injurious than carbonic acid. Quoting from *Hammond's Hygiene*, p. 168:—"When we enter a room in which many persons are contained, we are struck at once by the oppressive character of the air. That this is not altogether due to the presence of carbonic acid, is very apparent from the peculiar odor evolved. The same is true of a chamber in which one has slept, and which has not yet been purified by ventilation; or of a bed which has been lain in."

At St. Cloud, it was noticed, a few years ago, that there was an annual visitation of typhoid fever in connexion with doubling the number of soldiers in the barracks, on the annual visits of the King. (*Hammond's Hygiene*, p. 428.) Medical literature is full of such instances of the origin of disease in overcrowding, especially in court-rooms, when constructed, as they formerly were, without any reference to the necessity for fresh air to breathe.

It may not be always practicable to have the freedom of outdoor air, but it is now practicable for all sleepers, sick or well, in hospitals or private dwellings, to have air that is as good as outdoor air, and sometimes a great deal better.

Yours, Respectfully,
DAVID PRINCE.

21 *W. 12th St., New York, Jan.* 26.

NOTE.—A good description of Mr. LYMAN'S apparatus may be found in the *Scientific American* for Dec. 9th, 1865.

